# Diffusion-Weighted Lesions After Intracerebral Hemorrhage: Associated MRI Findings

**DOI:** 10.3389/fneur.2022.882070

**Published:** 2022-06-15

**Authors:** Kim Wiegertjes, Sabine Voigt, Wilmar M. T. Jolink, Emma A. Koemans, Floris H. B. M. Schreuder, Marianne A. A. van Walderveen, Marieke J. H. Wermer, Frederick J. A. Meijer, Marco Duering, Frank-Erik de Leeuw, Catharina J. M. Klijn

**Affiliations:** ^1^Department of Neurology, Donders Institute for Brain, Cognition and Behaviour, Radboud University Medical Center, Nijmegen, Netherlands; ^2^Department of Neurology, Leiden University Medical Center, Leiden, Netherlands; ^3^Department of Radiology, Leiden University Medical Center, Leiden, Netherlands; ^4^Department of Neurology and Neurosurgery, University Medical Center Utrecht, Brain Center, Utrecht University, Utrecht, Netherlands; ^5^Department of Medical Imaging, Radboud University Medical Center, Nijmegen, Netherlands; ^6^Department of Biomedical Engineering, Medical Image Analysis Center (MIAC AG) and qbig, University of Basel, Basel, Switzerland

**Keywords:** diffusion-weighted imaging, intracerebral hemorrhage, small vessel disease, magnetic resonance imaging, cerebral amyloid angiopathy

## Abstract

The current study aimed to investigate whether diffusion-weighted imaging-positive (DWI+) lesions after acute intracerebral hemorrhage (ICH) are associated with underlying small vessel disease (SVD) or linked to the acute ICH. We included patients ≥18 years with spontaneous ICH confirmed on neuroimaging and performed 3T MRIs after a median of 11 days (interquartile range [IQR] 6–43). DWI+ lesions were assessed in relation to the hematoma (perihematomal vs. distant and ipsilateral vs. contralateral). Differences in clinical characteristics, ICH characteristics, and MRI markers of SVD between participants with or without DWI+ lesions were investigated using non-parametric tests. We observed 54 DWI+ lesions in 30 (22%) of the 138 patients (median age [IQR] 65 [55–73] years; 71% men, 59 lobar ICH) with available DWI images. We found DWI+ lesions ipsilateral (54%) and contralateral (46%) to the ICH, and 5 (9%) DWI+ lesions were located in the immediate perihematomal region. DWI+ lesion presence was associated with probable CAA diagnosis (38 vs. 15%, *p* = 0.01) and larger ICH volumes (37 [8–47] vs. 12 [6–24] ml, *p* = 0.01), but not with imaging features of SVD. Our findings suggest that DWI+ lesions after ICH are a feature of both the underlying SVD and ICH-related mechanisms.

## Introduction

In patients with acute intracerebral hemorrhage (ICH) due to small vessel disease (SVD), diffusion-weighted imaging-positive (DWI+) lesions occur on average in 20% on MRI performed within 90 days (1). These DWI+ lesions have been associated with worse outcomes (2). Although DWI+ lesions are less frequent in patients with SVD without ICH (<5%) (3), it remains uncertain whether this high frequency of DWI+ lesions is secondary to the acute ICH or the result of the underlying SVD.

Recent meta-analyses on individual patient data demonstrated the association between DWI+ lesions after ICH with both magnetic resonance (MRI) markers of cerebral SVD and ICH characteristics, but data on the timing and location of DWI+ lesions in relation to the ICH were not available (1, 2). Therefore, we aimed to investigate the prevalence, timing, and location of DWI+ lesions in survivors of SVD-related ICH.

## Materials and Methods

Detailed methods are available in the online data supplement.

### Study Design and Population

A total of 204 patients presenting to the University Medical Centers of Nijmegen, Utrecht, or Leiden, with a symptomatic spontaneous ICH, confirmed on CT were included in the multi-center prospective cohort Finding the ETiology in spontaneous Cerebral Hemorrhage (FETCH) study between October 2013 and December 2018, of whom 155 underwent 3T MRI (4). Secondary causes of ICH were excluded by CT angiography, MRI/magnetic resonance angiography, or angiogram. There were minor differences in patient demographics and ICH characteristics on brain CT between the 155 participants who underwent MRI and 49 participants without MRI (Supplementary Table S1). Of all participants who had MRI, 17 participants were excluded due to inadequate DWI image quality. This resulted in a final sample size of 138 participants (median [IQR] age 65 [55–73] years; 98 men [71%]) with a median interval between the qualifying ICH and MRI of 11 days (IQR 6–43). In total, 112 patients underwent MRI in the acute phase (<7 days) and 26 in the non-acute phase (≥7 days). This study was approved by the Medical Ethics Review Committee of the University Medical Center Utrecht. Patients provided written informed consent.

### Neuroimaging Markers

Participants underwent 3T MRIs within 3 months of ICH onset using a standardized MRI protocol (Supplementary Table S2).

We classified the location of the ICH as lobar (cerebral lobes) or non-lobar (thalamus, basal ganglia, brainstem, or cerebellum) (5). We assessed ICH volume and perihematomal edema volume on FLAIR with manual segmentation using ITK-SNAP 3.8 (http://www.itksnap.org/). Subsequently, we calculated the edema extension distance (EED), as this measure is relatively independent of ICH volume (6). The following MRI markers of SVD were rated according to the STandards for ReportIng Vascular ChangEs on neuroimaging (STRIVE) criteria (7), using validated scales: white matter hyperintensities (WMH); lacunes; cerebral microbleeds (CMB); (8) any cortical superficial siderosis (focal and disseminated); (9) the probability of cerebral amyloid angiopathy according to the Modified Boston criteria (9).

Diffusion-weighted imaging-positive lesions were defined as hyperintense lesions ≤ 20 mm on diffusion-weighted imaging and a hypointense or isointense signal on apparent diffusion coefficient maps at the corresponding location and assessed by one trained rater (SV) (7). A second rater (EAK) assessed DWI+ lesion presence in a random subsample indicating perfect agreement (*n* = 10%, Cohen's kappa 1.00 [95% CI = 1–1]). The location of DWI+ lesions in relation to the ICH was assessed as follows: (1) perihematomal (<10 mm of the hematoma); (2) ipsilateral or contralateral; (3) supratentorial or infratentorial. DWI images and corresponding lesion masks were registered to the native T1-weighted images and subsequently normalized into MNI-152 standard space. Lesion coordinates were extracted from the co-registered lesion masks and visualized using BrainNet Viewer (http://www.nitrc.org/projects/nmv/) to investigate whether ipsilateral and contralateral DWI+ lesions have a similar distribution throughout the brain, which would be indicative of a more widespread cause than the acute ICH alone.

Diffusion-weighted data sets were pre-processed and diffusion tensors were estimated as described in detail in the online-only Data Supplement.

### Statistical Analysis

Statistical analyses were performed in R (R Foundation). Comparisons of DWI+ lesion prevalence between individuals with acute ( ≤ 7 days) vs. non-acute (>7 days) MRI scans and differences between individuals with or without any DWI+ lesions were performed using Mann-Whitney U tests and χ^2^ tests (or Fisher exact tests when appropriate). A two-tailed α was set at 0.05.

## Results

Among the 138 patients, 98 were men [71%], and their median age was 65 years (interquartile range [IQR] 55–73). In 59 of 138 patients, the ICH was lobar (43%), and in 79 (57%) non-lobar (56 deep and 23 infratentorial). Among the 59 patients, 24 (41%) with lobar ICH fulfilled the modified Boston criteria for probable CAA. Finally, 53 of the 138 patients had MRIs within 7 days.

**Figure 1:**
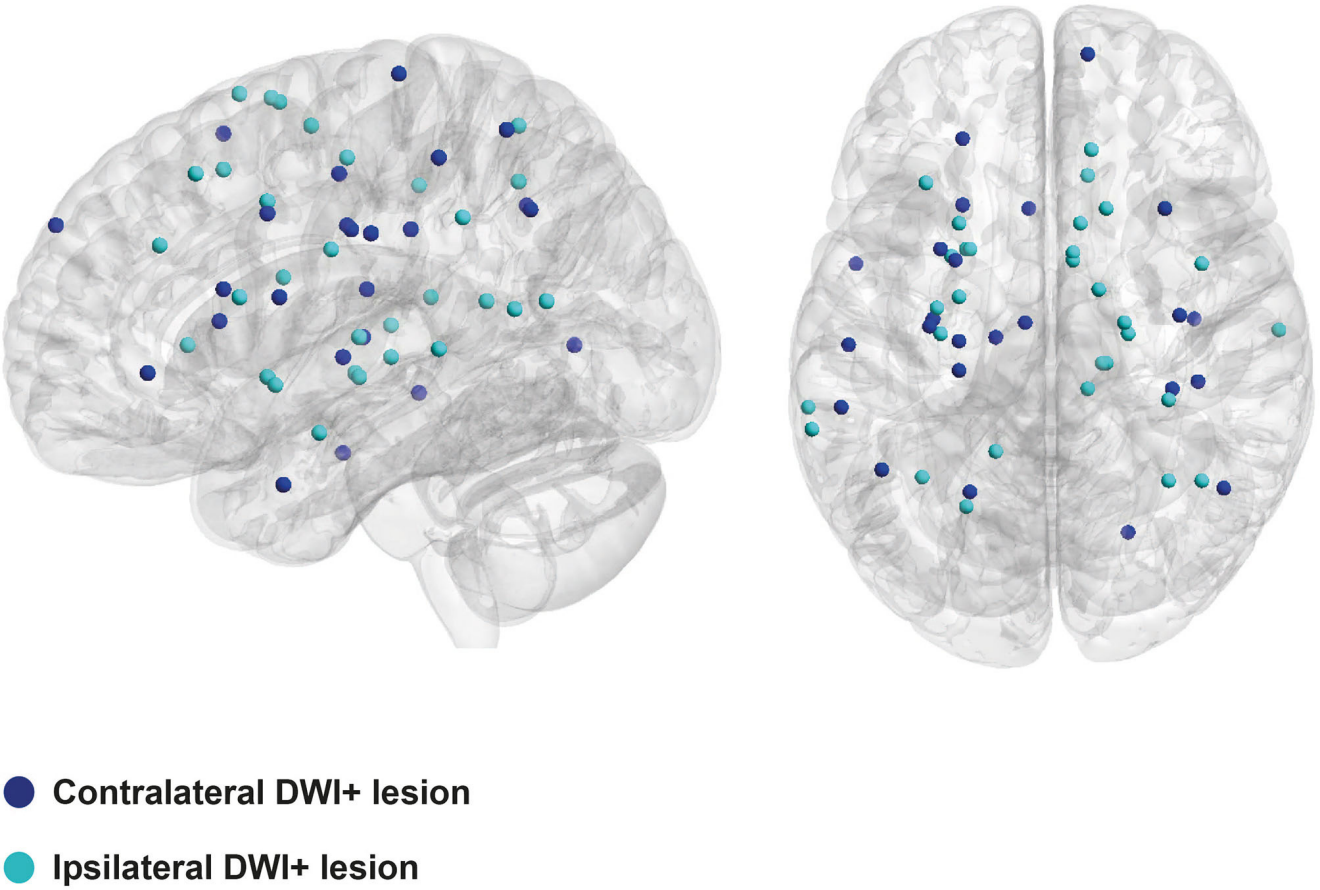
Topographical distribution of DWI+ lesions. DWI+, diffusion-weighted imaging-positive.

We identified 54 DWI+ lesions in 30 of the 138 patients (22%), of which 5 (9%) were located in perihematomal regions. DWI+ lesions were equally distributed in both hemispheres (ipsilateral 54% and contralateral 46%; Figure 1). Furthermore, supratentorial DWI+ lesions were not more frequent in patients with infratentorial ICH compared to patients with supratentorial ICH (26 vs. 20%, *p* = 0.50). There were more patients with probable CAA in the DWI+ group compared to the DWI- group (38 vs. 15%, *p* = 0.01). Individuals with probable CAA more often had strictly cortical DWI+ lesions than individuals with no-CAA ICH (78 vs. 17%, *p* < 0.01).

**Figure 2:**
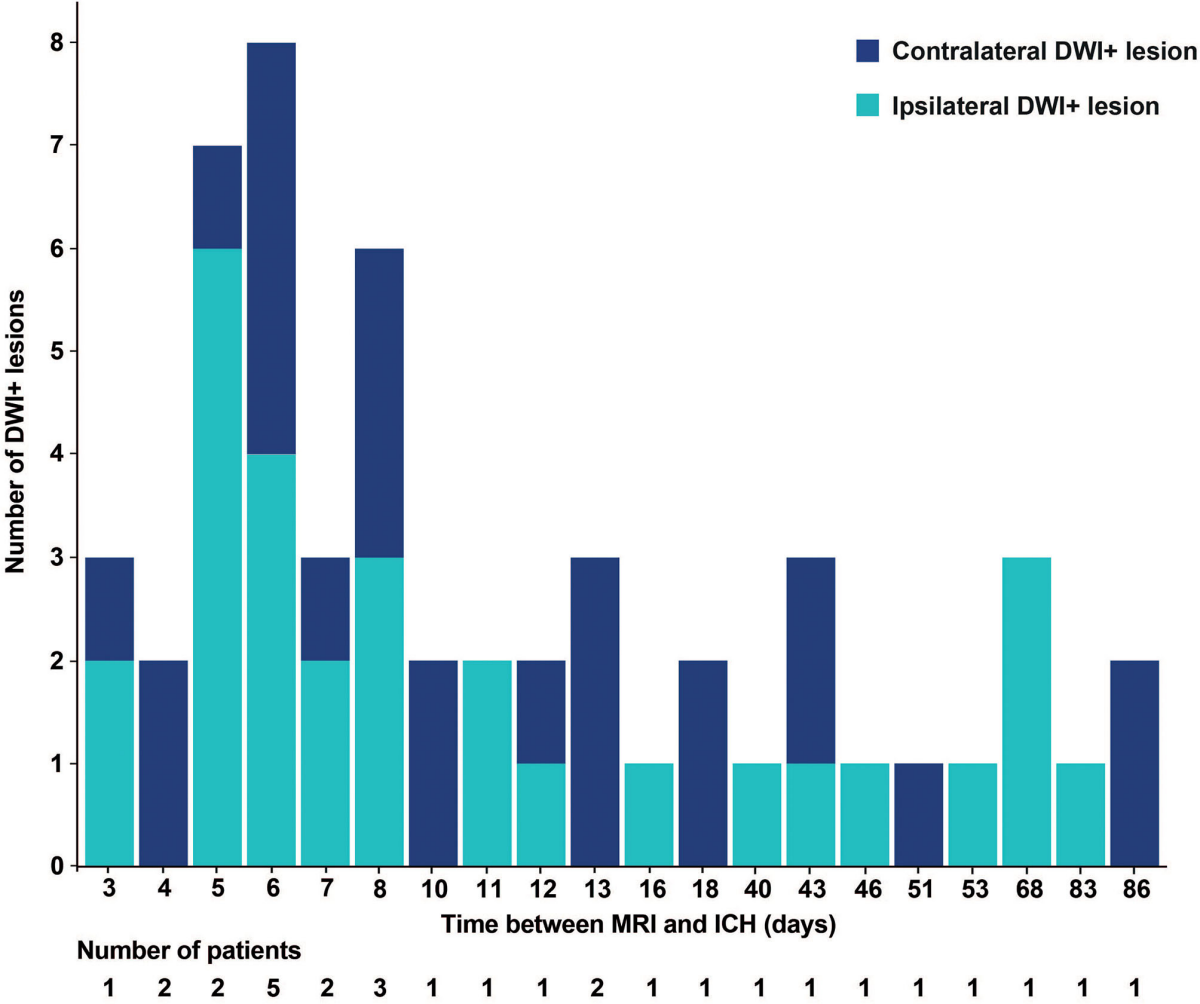
Histogram of the number of DWI+ lesions and time between MRI and ICH. DWI+, diffusion-weighted imaging-positive. MRI, magnetic resonance imaging; ICH, intracerebral hemorrhage.

We observed DWI+ lesions on MRIs up to 86 days after the ICH (Figure 2). The frequency of DWI+ lesions appeared similar on acute and non-acute MRI scans. In total, 12 of the patients with recent ICH had DWI+ lesions (23%) compared to 18 of the individuals with non-recent MRI (21%, *p* = 0.90).

Although we found larger ICH volumes in patients with DWI+ lesions relative to those without (37 [8–47] vs. 12 [6–24] ml, *p* = 0.01), clinical characteristics and SVD imaging features were similar between groups (Table 1).

**Table 1:** Characteristics of participants with and without DWI+ lesions.

	**No DWI+ lesion (*n* =108)**	**DWI+ lesion** **(*n* =30)**	***P***
**Clinical characteristics**			
Age, y	64 [53–72]	68 [61–75]	0.24
Men, *n* (%)	75 (69%)	23 (77%)	0.44
Hypertension	58 (54%)	21 (70%)	0.11
Diabetes	14 (13%)	5 (17%)	0.56
Hypercholesterolemia	35 (32%)	12 (40%)	0.44
BMI, kg/m^2^	26 [24–29]	25 [23–29]	0.62
Smoking, ever	59 (57%)	18 (67%)	0.35
Antithrombotic agents	45 (42%)	11 (37%)	0.62
**ICH characteristics**			
Side			0.12
Left	56 (53%)	11 (37%)	
Right	50 (47%)	19 (63%)	
Location of the ICH			0.19
Lobar	43 (40%)	16 (53%)	
Non-lobar	65 (60%)	14 (47%)	
ICH volume, mL	12 [6–24]	37 [8–47]	**0.01**
EED, cm	0.44 [0.22–0.61]	0.48 [0.29–0.60]	0.47
**MRI markers of SVD**			
WMH volume, % of ICV volume	0.33 [0.14–0.87]	0.61 [0.23–1.29]	*0.05*
Lacunes, prevalence	19 (18%)	5 (17%)	0.90
Microbleeds, prevalence	54 (55%)	19 (68%)	0.21
Cortical superficial siderosis, prevalence	14 (13%)	7 (26%)	0.15
MD, 10^−4^ mm^2^/s			*0.07*
Siemens	7.91 [7.62–8.16]	8.41 [7.99–8.80]	
Phillips	7.78 [7.46–8.07]	7.76 [7.59–7.98]	
Modified Boston Criteria			**0.01**
Probable CAA	14 (15%)	10 (38%)	
Other	81 (85%)	16 (62%)	

*Data are median (interquartile range) or number (%). DWI+ indicates patients with diffusion-weighted imaging-positive lesions; DWI-, patients without DWI+ lesions; ICH, intracerebral hemorrhage; ICV, intracranial volume; EED, edema extension distance; MRI, magnetic resonance imaging; BMI, body mass index; WMH, white matter hyperintensities; CAA, cerebral amyloid angiopathy; MD, mean diffusivity. Information on the history of smoking was missing in 8 (7%), presence of lacunes in 5 (5%), presence of microbleeds in 11 (8%), presence of cortical superficial siderosis in 10 (9%), and CAA diagnosis in 17 (16%) patients, due to incomplete information or poor image quality*.

## Discussion

Our results demonstrate a widespread distribution of DWI+ lesions after spontaneous ICH, unrelated to the anatomical location of the hematoma and unrelated to the time after ICH onset. DWI+ lesion presence was associated with ICH volume and a diagnosis of probable CAA, but not with all imaging features of SVD.

The similar distribution of ipsilateral and contralateral DWI+ lesions, and their high frequency outside the acute post-ICH period, suggest that these lesions are reflections of the underlying SVD. The absence of a statistically significant association between DWI+ lesions and SVD markers (other than those indicating CAA) could be due to the small sample size. The finding that DWI+ lesions were more frequent in cases diagnosed with probable CAA, especially in cortical regions, may indicate that the accumulation of amyloid-β in the vessel walls of cortical and leptomeningeal vessels could be related to the formation of abnormal blood clots resistant to degradation, as demonstrated in recent *in vitro* and *in vivo* studies (10). Future studies should investigate whether (cortical) DWI+ lesions are different between CAA vs. non-CAA patients and whether they have distinct underlying pathophysiological mechanisms.

Global mechanisms secondary to the ICH, such as immune-mediated mechanisms or blood-brain barrier breakdown, could also be involved in the origin of ICH-related DWI+ lesions (11), since patients with DWI+ lesions had considerably larger ICH volumes. Although beyond the scope of the current study, hypertension and its treatment in the acute phase may have influenced the occurrence of DWI+ lesions (12). However, the number of patients with hypertension did not differ between groups with and without DWI+ lesions. Furthermore, a recent individual patient data meta-analyses investigated ICH-related DWI+ lesions, blood pressure control, and outcomes, and did not find any associations (2), and a recent randomized controlled trial demonstrated that intensive acute blood pressure lowering did not lower the frequency of DWI+ lesions in ICH-patients (13), Alternatively, some hemorrhages might be caused by the hemorrhagic transformation of DWI+ lesions (14, 15).

Whereas, CAA is a major risk factor for recurrent ICH, this study suggests that CAA also predisposes to a higher DWI+ lesion frequency. This poses a clinical dilemma regarding the risk of future hemorrhagic or ischemic events when treating patients with acute ICH, including the use or avoidance of antithrombotic therapy or aggressive blood pressure lowering (16, 17), which should be addressed in future randomized controlled clinical trials.

The main strength of our study includes the unique cohort of ICH patients who underwent MRI during the acute or subacute phase of ICH. However, not all patients underwent MRI, in particular those who were in poor clinical condition, causing selection bias. Limitations of our study are the small sample size preventing us from multivariable regression analysis adjusted for several confounders, and the use of different scanners, although with aligned protocols.

These findings suggest that ICH-related DWI+ lesions are an ongoing process associated with the underlying SVD and are possibly promoted by the acute event of the ICH.

## Data Availability Statement

Data request can be sent to the corresponding author (karin.klijn@radboudumc.nl).

## Ethics Statement

The studies involving human participants were reviewed and approved by Medical Ethics Review Committee of the University Medical Center Utrecht. The patients/participants provided their written informed consent to participate in this study.

## Author Contributions

KW performed the statistical analysis, data interpretation, and drafting and revision of the manuscript. SV, WJ, EK, and FM participated in data acquisition and revised the manuscript for intellectual content. MWa, MWe, and FM contributed to the study concept and design and revised the manuscript for intellectual content. MD contributed to data interpretation and revised the manuscript for intellectual content. F-EL contributed to data interpretation and drafting and revision of the manuscript. CK contributed to the study design and concept, data acquisition and interpretation, and drafting and revision of the manuscript. All authors contributed to the article and approved the submitted version.

## Funding

MWe was supported by a personal ZonMw VIDI grant (91717337), the Netherlands Heart Foundation (2016T086), and the Dutch CAA Foundation. MD was supported by the German Research Foundation (DU1626/1–1). FS was supported by the Dutch Heart Foundation (Grant No. 2019T060). FS and CK were supported by a clinically established investigator grant from the Dutch Heart Foundation (Grant No. 2012 T077). CK was supported by an Aspasia grant from The Netherlands Organization for Health Research and Development (ZonMw Grant No. 015.008.048). F-ED was supported by a clinically established investigator grant of the Dutch Heart Foundation (Grant No. 2014 T060) and by a VIDI innovational grant from the Netherlands Organization for Health Research and Development (ZonMw Grant No. 016.126.351).

## Conflict of Interest

The authors declare that the research was conducted in the absence of any commercial or financial relationships that could be construed as a potential conflict of interest.

## Publisher's Note

All claims expressed in this article are solely those of the authors and do not necessarily represent those of their affiliated organizations, or those of the publisher, the editors and the reviewers. Any product that may be evaluated in this article, or claim that may be made by its manufacturer, is not guaranteed or endorsed by the publisher.
