# Supplementary material for: Diffusion-Weighted Lesions After Intracerebral Hemorrhage: Associated MRI Findings
Source: Front Neurol. 2022 Jun 15;13:882070. doi: 10.3389/fneur.2022.882070 (PMC9240258; doi:10.3389/fneur.2022.882070)
Supplement: Supplementary file 1 [file Table_1.DOCX]

**Supplementary Material**

Diffusion-weighted lesions after intracerebral hemorrhage: associated MRI findings

**Supplemental Methods**

**Supplemental References**

**Supplemental Table 1:** Characteristics of participants with and without 3T brain MRI
**Supplemental Table 2:** MRI parameters

**Supplemental Methods**

We pre-processed diffusion-weighted data sets to correct for eddy current-induced distortions and head motion, using tools from Mrtrix3 (httpp://www.mrtrix.org/, dwidenoise, mrdegibbs) and the Functional Magnetic Resonance Imaging of the Brain (FMRIB) Software Library (FSL; version 6.0.1, eddy).^1^ After pre-processing, trace images were calculated based on the geometric mean of all DWI volumes, using MATLAB (version R2019a, The MathWorks, Inc). Diffusion tensors were estimated using linear least squares implemented in Matlab. DTI measures, such as mean diffusivity were calculated from the tensors for each voxel using fslmaths, part of FSL. A white matter skeleton was obtained using Tract-Based Spatial Statistics from FSL, using a FA threshold of > 0.2 and a custom-made mask,^2^ excluding areas susceptible to contamination by cerebrospinal fluid. The mean of all voxels of the hemisphere contralateral to the ICH located in the masked skeleton were used for further analysis. Since diffusion data collected from different MRI scanners can vary significantly even when using a harmonious MRI protocol,^3^ we stratified our analysis according to the type of MRI scanner (see eTable 1).

**Supplemental References**

1. Smith SM, Jenkinson M, Woolrich MW, Beckmann CF, Behrens TE, Johansen-Berg H, Bannister PR, De Luca M, Drobnjak I, Flitney DE, et al. Advances in functional and structural mr image analysis and implementation as fsl. *Neuroimage*. 2004;23:208-219

2. Baykara E, Gesierich B, Adam R, Tuladhar AM, Biesbroek JM, Koek HL, Ropele S, Jouvent E, Chabriat H, Ertl-Wagner B, et al. A novel imaging marker for small vessel disease based on skeletonization of white matter tracts and diffusion histograms. *Annals of Neurology*. 2016;80:581-592

3. Mirzaalian H, Ning L, Savadjiev P, Pasternak O, Bouix S, Michailovich O, Karmacharya S, Grant G, Marx CE, Morey RA, et al. Multi-site harmonization of diffusion mri data in a registration framework. *Brain Imaging Behav*. 2018;12:284-295

**Table 1.** Characteristics of participants with and without brain MRI

|  | **No MRI (n=49)** | **MRI (n=155)** | **P** |
| --- | --- | --- | --- |
| **Clinical characteristics** |  |  |  |
| Age, y | 72 [61-80] | 65 [56-74] | **.02** |
| Men | 29 (59%) | 111 (71%) | .14 |
| Hypertension | 29 (59%) | 86 (55%) | .45 |
| Diabetes | 3 (6%) | 24 (15%) | .11 |
| Hypercholesterolemia | 16 (33%) | 52 (34%) | .95 |
| BMI, kg/m^2^ | 26 [23-28] | 26 [23-29] | .80 |
| Smoking, ever | 26 (53%) | 87 (56%) | .88 |
| Antithrombotic agents | 23 (47%) | 63 (41%) | .33 |
| **ICH characteristics** |  |  |  |
| Side |  |  | .08 |
| Left | 29 (59%) | 74 (31%) |  |
| Right | 17 (35%) | 79 (51%) |  |
| Location of the ICH |  |  | **<.01** |
| Lobar | 9 (18%) | 70 (45%) |  |
| Non-lobar | 38 (78%) | 85 (55%) |  |

Data are median (interquartile range) or number (%). MRI, magnetic resonance imaging; BMI, body mass index; ICH, intracerebral hemorrhage.

**Table 2.** MRI protocol

| **Sequence** |  | **Utrecht** | **Leiden** | **Nijmegen** |
| --- | --- | --- | --- | --- |
|  | **MRI scanner*** | **Phillips** | **Phillips** | **Siemens** |
|  | Patients [n] | 64 | 33 | 41 |
| T1 | Slice [mm] | 1.00 | 1.00 | 1.00 |
|  | In-plane [mm] | 1.00 | 1.00 | 1.00 |
| FLAIR | Slice [mm] | 3.00 | 3.00 | 3.00 |
|  | In-plane [mm] | 0.96 | 0.96 | 0.96 |
| DWI | Slice [mm] | 2.50 | 2.50 | 2.00 |
|  | In-plane [mm] | 1.72 | 1.72 | 2.00 |
|  | Diffusion directions | 45 | 45 | 64 |
| SWI | Slice [mm] | - | 3.00 | - |
|  | In-plane [mm] | - | 0.96 | - |
| T2* | Slice [mm] | 3.00 | - | 3.00 |
|  | In-plane [mm] | 0.98 | - | 0.98 |

MRI, magnetic resonance imaging; FLAIR, fluid-attenuated inversion recovery; DWI, diffusion-weighted imaging; SWI, susceptibility-weighted imaging.

***** different MRI scanners (Siemens Healthineers, Erlangen, Germany; Phillips Healthcare, Best, The Netherlands)
